# Rhodium(I)-Complexes Catalyzed 1,4-Conjugate Addition of Arylzinc Chlorides to *N*-Boc-4-pyridone

**DOI:** 10.3390/molecules22050723

**Published:** 2017-05-01

**Authors:** Fenghai Guo, Matthew A. McGilvary, Malcolm C. Jeffries, Briana N. Graves, Shekinah A. Graham, Yuelin Wu

**Affiliations:** 1 Department of Chemistry, Winston-Salem State University, Winston Salem, NC 27110, USA; mcgilvary336@gmail.com (M.A.M.); mjeffries115@rams.wssu.edu (M.C.J.); bgraves114@rams.wssu.edu (B.N.G.); sgraham113@rams.wssu.edu (S.A.G.); 2 School of Chemical and Environmental Engineering, Shanghai Institute of Technology, Shanghai 201418, China; wuyuelin517@hotmail.com

**Keywords:** *N*-boc-4-pyridone, rhodium (I)-complexes, conjugate addition, 2,3-dihydropyridones

## Abstract

Rhodium(I)-complexes catalyzed the 1,4-conjugate addition of arylzinc chlorides to *N*-Boc-4-pyridone in the presence of chlorotrimethylsilane (TMSCl). A combination of [RhCl(C_2_H_4_)_2_]_2_ and BINAP was determined to be the most effective catalyst to promote the 1,4-conjugate addition reactions of arylzinc chlorides to *N*-Boc-4-pyridone. A broad scope of arylzinc reagents with both electron-withdrawing and electron-donating substituents on the aromatic ring successfully underwent 1,4-conjugate addition to *N*-Boc-4-pyridone to afford versatile 1,4-adducts 2-substituted-2,3-dihydropyridones in good to excellent yields (up to 91%) and excellent ee (up to 96%) when (*S*)-BINAP was used as chiral ligand.

## 1. Introduction

1,4-conjugate addition of organometallic reagents to enones is one of the most reliable and widely used carbon-carbon bond formation processes to afford β-substituted carbonyl compounds, which are highly useful synthons for further organic transformations [1,2,3,4,5]. The combination of metal catalysts with an organometallic reagent has been particularly effective in promoting 1,4-addition to enones. Among the metal catalysts, copper is the most commonly used species [3]. Other metal catalysts including nickel and palladium have also been reported to be effective in 1,4-conjugate addition of organozinc, organoaluminum, organoziconium, and organomercury compounds to α,β-unsaturated enones. Organometallic reagents such as organolithiums, Grignard reagents and diorganozinc reagents have also been widely used in this regard and high yields of 1,4-adducts can be achieved in most cases. However, the competing 1,2-additions as well as 1,6-additions accompanied with these organometallic reagents have limited their applications [6,7,8,9,10,11]. Over the last 30 years, significant progresses have been made in asymmetric 1,4-conjugate additions, especially in the addition of organozinc or Grignard reagents using copper(I) catalysts in combination with chiral phosphorous ligands [12,13,14]. Although high yields and high entioselectivity can be achieved in these copper-catalyzed reactions, the substituents introduced to the β-position are limited to alkyl groups [12,13,14]. In recent years, there has been a growing interest in rhodium-catalyzed C-C bond forming reactions of organometallic reagents, due to the mild reaction conditions and toleration of various functional groups [15,16]. Since the first report by Miyaura in 1997 [17], rhodium-catalyzed 1,4-conjugate addition of organometallic reagents to unsaturated substrates has emerged as a versatile and efficient methodology for the formation of C-C bonds due to their tolerance with water and a wide range of substrates [18,19]. Rhodium-catalyzed 1,4-conjugate addition reactions utilize mild organometallic reagents such as organoboron and arylzinc reagents under mild reaction conditions, which are extremely useful in introducing versatile aromatic groups to the β-position. For the abovementioned reasons, considerable efforts have been devoted to developing the rhodium-catalyzed 1,4-conjugate addition of alkenyl(aryl)boronic acids as well as arylzinc reagents. A broad scope of substrates such as cyclic or acyclic enones and enoates have been reported [18,19]. Rhodium(I) complexes have been demonstrated to be excellent catalysts for 1,4-conjugate addition of alkenyl- and arylboronic acids to α,β-unsaturated ketones, esters, and even less reactive amides [18,19,20,21,22,23,24,25,26,27].

**Scheme 1:**
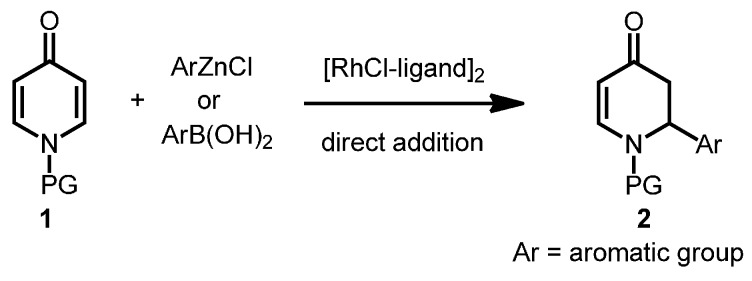
Rh(I)-catalyzed C-C bond formation.

In the course of our investigation on access to highly versatile 2-substituted-2,3-dihydropyridones **2**, we are intrigued by the possibility of rhodium(I)-complex catalyzed 1,4-addition of arylzinc cholorides or arylboronic acids to *N*-protected-4-pyridones **1** (Scheme 1). We are interested in *N*-heterocycles such as 2-substituted-2,3-dihyrdopyrdones **2** because they are an important class of medicinal compounds, and many medicinally important compounds in clinical or pre-clinical studies contain piperidine subunits [28,29,30,31]. 2-Substituted-2,3-dihyrdopyrdines are precursors for medicinally important *N*-heterocycles such as pyridines, piperidones, piperidines, indolizidenes, and quinolizidenes.

Due to their importance in medicinal chemistry, considerable efforts have been devoted to develop efficient synthetic strategies for the synthesis of piperidine derivatives. Most of these strategies involve using chiral auxiliaries/chiral starting materials and have been developed into effective methods for the synthesis of a wide variety of *N*-heterocycles [32,33,34,35,36,37,38,39,40]. The synthesis of 2,3-dihydro-4-pyridones via conjugate addition reactions have also been reported [38,39,40,41,42,43,44,45,46,47,48,49,50,51]. Great progress has been made using dialkylzinc reagents in conjunction with copper catalysts, but challenges still remain in increasing reactivity and general applicability [40,41,42,43,44,45,46]. Recent developments in copper-catalyzed asymmetric conjugate additions of Grignard reagents [47,50,51] and rhodium-catalyzed 1,4-addition of arylboroxines/boronic acids promise greater reactivity and versatility [40]. Recent advances in the NHC-Cu-catalyzed conjugate arylation of β-substututed cyclic enones to afford quaternary stereogenic centers has also been reported [52]. Despite the fact that rhodium(I)-complexes-catalyzed 1,4-conjugate addition of boronic acids is one of the most potent methods for C-C bond formation, there are no reported examples on 1,4-conjugate addition of boronic acids to *N*-Boc-4-pyridones or substrates that contain piperidine subunits, presumably due to the unreactive nature of these nitrogen-containing substrates with boronic acids [20,21,22,23,24,25,26,27].

## 2. Results and Discussions

**Scheme 2:**
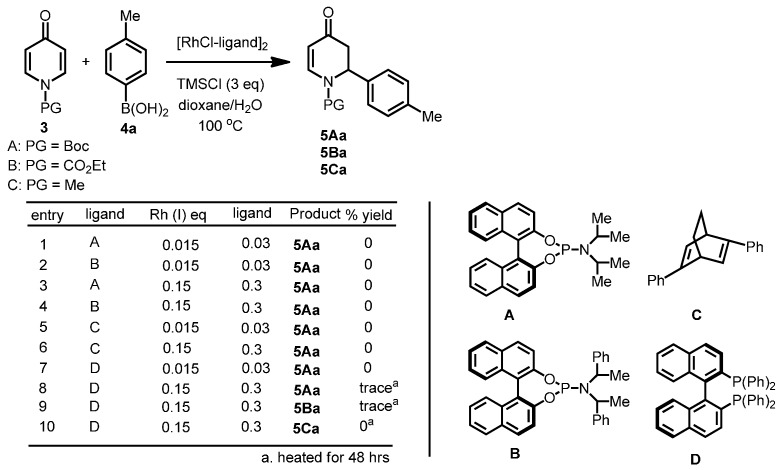
Rh-catalyzed conjugate addition of arylboronic acid.

As a starting point, we employed the standard rhodium(I)-BINAP reaction conditions (Scheme 2) [20,53]. Initially, the reaction of *N*-Boc-4-pyridone **3A** with 2.0 equiv of *p*-tolylboronic acid **4a** was carried out in dioxane/water (10 to 1 ratio) in the presence of 1.5 mol % of [RhCl(C_2_H_4_)_2_]_2_ and 3 mol % of ligands such as phosphoramidite ligands (A, B) [12,13,14], 1,5-diphenyl-1,5-cyclooctadiene (C) [54], and BINAP (D) [18,19]. Under these standard reactions conditions, no 1,4-adduct **5Aa** was formed (Scheme 2, entries 1–2, 5, 7). No desired 1,4-adduct **5Aa** was formed even after 24 h at 100 °C in dioxane/water (10 to 1 ratio) with the increased catalyst loading of 15 mol % of [RhCl(C_2_H_4_)_2_]_2_ and 30 mol % of ligands (Scheme 2, entries 3–4, 6). Only a trace amount of 1,4-adduct **5Aa** was observed after extended heating with 15 mol % [RhCl(C_2_H_4_)_2_]_2_ and 30 mol % of BINAP at 100 °C for 48 h (Scheme 2, entry 8). The substituents on nitrogen were also investigated (Scheme 2, entries 9–10). With ethyl carbonate as a protecting group, a trace amount of 1,4-adduct **5Ba** was observed. No 1,4-adduct **5Ca** was attained with N-methylated substrate **3C** (Scheme 2, entry 10).

**Scheme 3:**
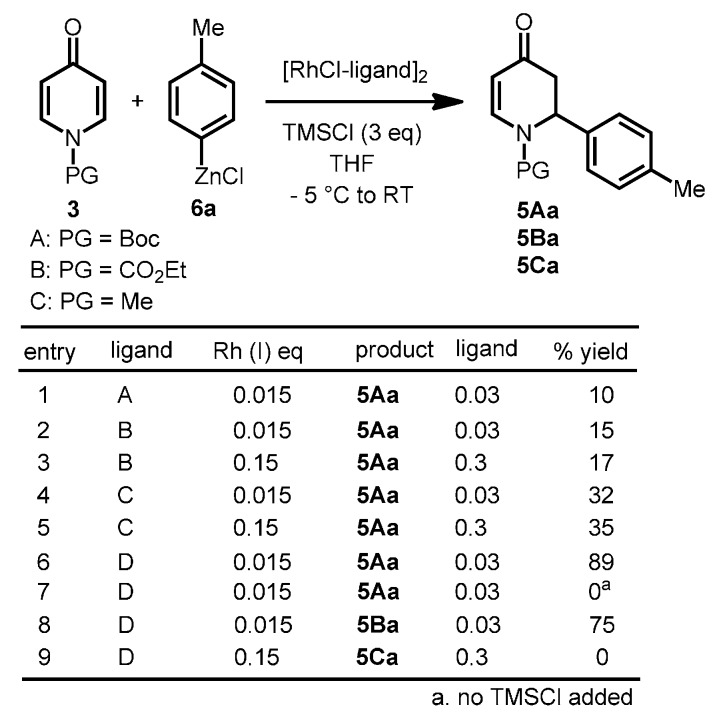
Optimization of Rh-catalyzed conjugate addition.

We then turned our attention to arylzinc chlorides, which have been reported to be more reactive nucleophiles towards 1-benzyloxycarbonyl-4-quinolone and structurally similar 2,3-dihydro-4-pyridones under rhodium(I)-complexes-catalyzed reaction conditions [18,19]. There is also one isolated example on rhodium(I)-BINAP-catalyzed 1,4-conjugate addition to *N*-*tert*-butoxycarbonyl-4-pyridone [55]. Other approaches involving 2,3-dihydro-4-pyridones via direct conjugate addition of organocuprates and Grignard reagents have also been reported [56,57]. In our study, ligands such as phosphoramidite ligands (A, B) [12,13,14], 1,5-diphenyl-1,5-cyclooctadiene (C) [54], and BINAP (D) [18,19] were initially studied in rhodium(I)-catalyzed conjugate addition of p-tolylZnCl **6a** to *N*-Boc 4-pyridone **3A** at −5 °C. As shown in Scheme 3, p-tolylZnCl **6a** underwent 1,4-conjugate addition to *N*-Boc-4-pyridone **3A** catalyzed by rhodium(I)-phosphoramidite in the presence of chlorotrimethyl silane (TMSCl) with low chemical yield (Scheme 3, entry 1). Slightly higher chemical yield was observed when phosphoramidite B was used as a ligand (Scheme 3, entry 2). When 1,5-diphenyl-1,5-cyclootadiene was used with [RhCl(C_2_H_4_)_2_]_2_, a higher chemical yield 32% can be attained under similar reaction conditions (entry 4). With both ligands A and B, no significant increases in chemical yields were obtained, even with 15 mol % [RhCl(C_2_H_4_)_2_]_2_ and 30 mol % of ligands (Scheme 3, entries 3 and 5). This ligand screening showed that when 3 mol % of BINAP was used as a ligand, 1,4-adduct **5Aa** can be attained in good chemical yield (89%, Scheme 3, entry 6). We also investigated the effect of substituents on nitrogen (Scheme 3, entries 8–9). With ethyl carbonate as a protecting group, the yield of 1,4-adduct **5Ba** was much lower (Scheme 3, entry 8). No 1,4-adduct **5Ca** was attained with N-methylated substrate **3C** (Scheme 3, entry 9). Notably no desired 1,4-adduct was observed without the addition of TMSCl (Scheme 3, entry 7). It has been reported that the addition of chlorotrimethylsilane as a Lewis acid may facilitate the activation of the substrate toward 1,4-addition and also stabilize the product by forming a silyl enol ether, which is then converted to the carbonyl group under acidic work-up conditions [58].

**Scheme 4:**
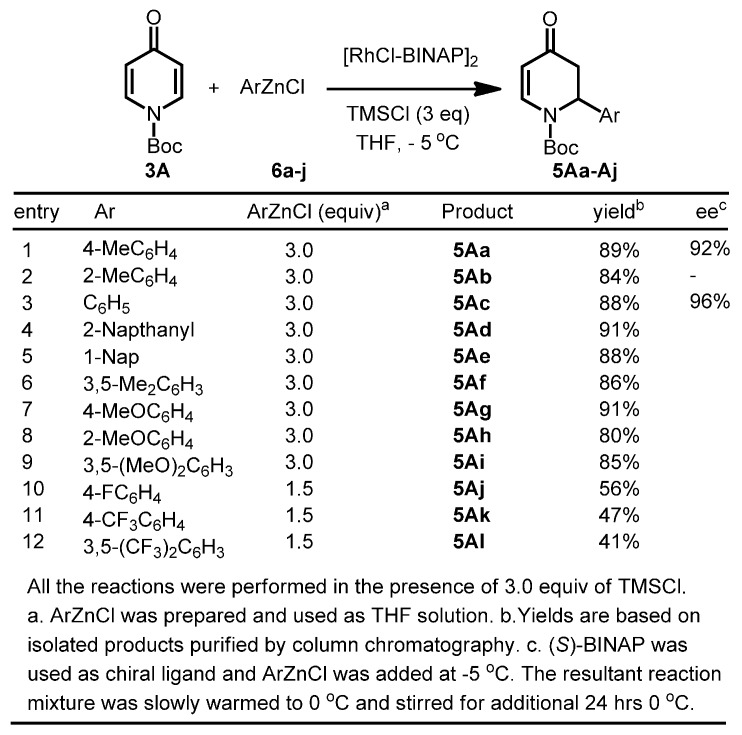
Rh(I)-BINAP-catalyzed conjugate addition reaction.

With the optimized reaction conditions in hand, we next examined the scope of rhodium-BINAP-catalyzed arylzinc reagents conjugate addition (Scheme 4). In general, arylzinc reagents underwent smooth conjugate addition to *N*-Boc-4-pyridone with good to excellent chemical yields. Simple arylzinc reagents such as phenylzinc and naphthylzinc chlorides added to *N*-Boc-4-pyridone with excellent chemical yields (Scheme 4, entries 3–5). The arylzinc reagents with electron donating substituents usually showed higher reactivity (Scheme 4, entries 1, 2, 6–9). The arylzinc reagents with strong electron withdrawing groups such as fluoro, trifluoromethyl, and bistrifluromethyl groups also underwent conjugate addition but with lower chemical yields (entries 10–12). Compared to *para*- and *meta*-substituted arylzinc reagents, the ortho-substituted arylzinc reagents gave lower yields due to steric hindrance (entries 2, 8). We also conducted asymmetric conjugate addition of arylzinc reagents to *N*-Boc-4-pyridone. When (*S*)-BINAP was used as the chiral ligand, excellent ee can be achieved (entries 1 and 3, up to 96% ee).

## 3. Materials and Methods

### 3.1. General Procedures, Materials and Intrumentation

The ^1^H- and ^13^C-NMR spectra were recorded on a BRUKER 300 NMR spectrometer (BRUKER, Winston Salem, NC, USA), operating at 300 MHz for ^1^H, 75 MHz for ^13^C and 282 MHz for ^19^F. Samples for NMR spectra were dissolved in deuterated chloroform (with TMS). Infrared (IR) spectra were recorded on a Nicolet iS10 FT-IR spectrometer as neat samples (thin films). Analytical thin layer chromatography (TLC) was performed on silica gel plates, 60 µ mesh with F_254_ indicator. Visualization was accomplished by UV light (254 nm), and/or a 10% ethanol solution of phosphomolybdic acid and/or KMnO_4_ stain prepared by dissolving 1.5 g KMnO_4_, 10 g potassium carbonate, and 1.25 mL 10% sodium hydroxide in 200 mL water. Flash chromatography was performed with 200–400 µ silica gel.

#### 3.1.1. Materials

Unless stated otherwise, solvents and chemicals were obtained from commercial sources and used without further purification. Anhydrous tetrahydrofuran (THF) was purchased from Sigma Aldrich. TMSCl was distilled from CaH_2_ under a positive nitrogen atmosphere. Arylzinc reagents were prepared from the corresponding aryllithium reagent and ZnCl_2_. Aryllithium reagents were prepared from the corresponding arylhalides and *t*-BuLi (1.70 M in pentane). *t*-BuLi (1.70 M in pentane) was commercially available and titrated using *sec*-BuOH and 1,10-phenanthroline monohydrate in THF. All glassware was flamed-dried under high vacuum and purged with argon and then cooled under a dry nitrogen atmosphere. Low temperature baths were prepared using ice NaCl water bath, or dry ice-isopropanol slush bath mixtures. All ArZnCl 1,4-conjugate addition reactions were conducted under a positive, dry argon atmosphere in anhydrous solvents in flasks fitted with rubber septa.

#### 3.1.2. General Procedure A

Rh(I)-BINAP-catalyzed 1,4-conjugate addition reactions. This method was modified from the procedure reported by Hayashi [18]. Starting *N*-Boc-4-pyridone (0.5 mmol) was added to a solution of [RhCl(C_2_H_4_)_2_]_2_ (0.015 mol %, 0.0075 mmol) and BINAP (0.033 mol %, 0.0165 mmol) in dry THF (1.0 mL) at −5 °C under argon with continuous stirring. After stirring for 15 min, ArZnCl (1.0 M in THF, 3.0 quiv, 1.5 mmol, prepared from corresponding arylbromides in a separate flask [59]) and TMSCl (1.0 M in THF, 3.0 equiv, 1.5 mmol) were added simultaneously dropwise over 10 min to this solution. The resulting mixture was then warmed up to room temperature and stirred for 20 h at room temperature. Then, the reaction mixture was diluted with dichloromethane (4.0 mL), quenched with saturated aqueous NH_4_Cl (4.0 mL) and extracted with dichloromethane (3 × 8.0 mL). The combined organic phase was washed with water (8.0 mL), brine (8.0 mL), then dried over anhydrous Na_2_SO_4_, filtered, concentrated in vacuo, and purified by flash column chromatography (silica, 10%–20% ethyl acetate in hexanes, *v/v*) to give pure compounds.

HRMS data for compounds **5Aa**, **5Ad**, **5Af**−**Ag**, **5Ai**, **5Ak**–**Al** were analyzed by TOF MS, see supplementary. Compounds **5Ab**–**Ac**, **5Ae**, **5Ah**, and **5Aj** have been fully characterized and reported [55,57,60].

### 3.2. Synthesis of Adducts ***5Aa, 5Ad, 5Af–Ag, 5Ai, 5Ag, 5Ak–Al***

*N-Boc-2-(4-methylphenyl)-2,3-dihydro-4-pyridone* (**5Aa**). Employing General Procedure A and using *N*-Boc-4-pyridone (97 mg, 0.5 mmol), [RhCl(C_2_H_4_)_2_]_2_ (2.92 mg, 0.0075 mmol), (*S*)-BINAP (10.3 mg, 0.0165 mmol), ArZnCl (1.0 M in THF, 3.0 equiv, 1.5 mmol), TMSCl (163 mg, 1.5 mmol) and the resultant reaction mixture was slowly warmed to 0 °C and stirred for an additional 24 h at 0 °C, after purification by flash column chromatography (silica, 10%–20% ethyl acetate:hexanes, *v/v*) gave white solid **5Aa** (128 mg, 89%). The ee was determined on a Daicel Chiralcel OD-H column with a solvent system of hexanes/2-propanol (9:1 ratio), flow rate = 1.0 mL/min. t_r_(major) = 7.1 min., t_r_(minor) = 7.8 min. 92% ee. m.p. 82.6–84.3 °C; IR (neat) 3081 (w), 2982 (w), 2926 (w), 1729 (s), 1661 (s), 1597 (s), 1412 (w), 1369 (m), 1339 (s), 1299 (s), 1253 (m), 1210 (m), 1145 (s), 1096 (m), 1010 (m), 923 (m), 845 (m), 815 (m), 774 (m) cm^−1^; ^1^H-NMR δ 1.33 (s, 9 H), 2.17 (s, 3H), 2.63 (td, *J* = 1.5, 16.5 Hz, 1 H), 2.98 (dd, *J* = 7.50, 16.5 Hz, 1 H), 5.20 (dd, *J* = 1.17, 8.4 Hz, 1 H), 5.49 (d, *J* = 7.50 Hz, 1 H), 6.96 (s, 4 H), 7.79 (d, *J* = 8.4 Hz, 1 H); ^13^C-NMR δ 21.0, 28.0, 41.9, 55.4, 83.6, 107.0, 125.8, 129.4, 135.8, 137.6, 142.9, 151.5, 192.3. HRMS (EI-ion trap) *m/z*: [M]^+^ calcd for C_17_H_21_NO_3_, 287.1520; found 287.1521.

*N-Boc-2-(2-napthanyl)-2,3-dihydro-4-pyridone* (**5Ad**). Employing General Procedure A and using *N*-Boc-4-pyridone (97 mg, 0.5 mmol), [RhCl(C_2_H_4_)_2_]_2_ (2.92 mg, 0.0075 mmol), BINAP (10.3 mg, 0.0165 mmol), ArZnCl (1.0 M in THF, 3.0 equiv, 1.5 mmol) and TMSCl (163 mg, 1.5 mmol) after purification by flash column chromatography (silica, 10%–20% ethyl acetate:hexanes, *v/v*) gave white solid **5Ad** (147 mg, 91%): m.p. 113.8–115.1 °C; IR (neat) 3065 (w), 2981 (w), 1717 (s), 1667 (s), 1606 (s), 1451 (m), 1369 (m), 1309 (s), 1257 (m), 1222 (m), 1142 (s), 950 (m), 851 (m), 757 (s) cm^−1^; ^1^H-NMR (300 MHz, CDCl_3_) δ 1.40 (br s, 9H), 2.84 (d, *J* = 16.5 Hz, 1H), 3.16 (dd, *J* = 7.8, 16.5 Hz, 1H), 5.34 (d, *J* = 8.4 Hz, 1H), 5.76 (d, *J* = 7.2 Hz, 1H), 7.30 (dd, *J* = 1.5, 8.4 Hz, 1H), 7.35-7.43 (m, 2H), 7.68-7.76 (m, 3H), 7.97 (d, *J* = 8.4 Hz, 1H); ^13^C-NMR (75 MHz, CDCl_3_) δ 28.0, 41.8, 55.9, 83.8, 107.1, 123.9, 124.6, 126.2, 126.4, 127.6, 128.0, 128.9, 132.9, 133.2, 136.2, 142.9, 151.5, 192.0. HRMS (EI-ion trap) *m/z*: [M]^+^ calcd. for C_20_H_21_NO_3_, 323.1521; found 323.1529.

*N-Boc-2-(3,5-dimethylphenyl)-2,3-dihydro-4-pyridone* (**5Af**). Employing General Procedure A and using *N*-Boc-4-pyridone (97 mg, 0.5 mmol), [RhCl(C_2_H_4_)_2_]_2_ (2.92 mg, 0.0075 mmol), BINAP (10.3 mg, 0.0165 mmol), ArZnCl (1.0 M in THF, 3.0 equiv, 1.5 mmol) and TMSCl (163 mg, 1.5 mmol) after purification by flash column chromatography (silica, 10%–20% ethyl acetate:hexanes, *v/v*) gave white solid **5Af** (129 mg, 86%): m.p. 100.0–101.7 °C; IR (neat) 3012 (w), 2978 (w), 2918 (w), 1713 (s), 1663 (s), 1596 (s), 1460 (w), 1418 (m), 1369 (m), 1337 (m), 1315 (s), 1257 (m), 1220 (m), 1142 (s), 1017 (m), 843 (s), 762 (s), 703 (w) cm^−1^; ^1^H-NMR (300 MHz, CDCl_3_) δ 1.32 (br s, 9H), 1.13 (s, 6H), 2.64 (td, *J* = 1.5, 16.5 Hz, 1H), 2.98 (dd, *J* = 7.8, 16.5 Hz, 1H), 5.21 (dd, *J* = 1.2, 8.4 Hz, 1H), 5.44 (d, *J* = 7.8 Hz, 1H), 6.65 (s, 2H), 6.75 (s, 1H), 7.82 (d, *J* = 8.4 Hz, 1H); ^13^C-NMR (75 MHz, CDCl_3_) δ 21.4, 28.0, 41.9, 55.6, 83.6, 107.0, 123.5, 128.2, 129.0, 129.5, 138.3, 138.8, 143.0, 151.5, 192.3. HRMS (EI-ion trap) *m/z*: [M]^+^ calcd for C_18_H_23_NO_3_, 301.1678; found 301.1683.

*N-Boc-2-(4-methoxylphenyl)-2,3-dihydro-4-pyridone* (**5Ag**). Employing General Procedure A and using *N*-Boc-4-pyridone (97 mg, 0.5 mmol), [RhCl(C_2_H_4_)_2_]_2_ (2.92 mg, 0.0075 mmol), BINAP (10.3 mg, 0.0165 mmol), ArZnCl (1.0 M in THF, 3.0 equiv, 1.5 mmol) and TMSCl (163 mg, 1.5 mmol) after purification by flash column chromatography (silica, 10%–20% ethyl acetate:hexanes, *v/v*) gave white amorphous solid **5Ag** (138 mg, 91%): m.p. 70.0−71.9 °C; IR (neat) 2984 (w), 2932 (w), 2836 (w), 1719 (s), 1663 (s), 1594 (s), 1508 (s), 1508 (w), 1366 (w), 1342 (s), 1285 (s), 1246 (s), 1171 (s), 1104 (s), 1027 (s), 981 (m), 852 (w), 787 (m), 769 (s), 645 (m) cm^−1^; ^1^H-NMR (300 MHz, CDCl_3_) δ 1.47 (br s, 9H), 2.74 (td, *J* = 1.5, 16.5 Hz, 1H), 3.11 (dd, *J* = 7.5, 16.5 Hz, 1H), 3.77 (s, 3H), 5.34 (dd, *J* = 1.5, 8.4 Hz, 1H), 5.61 (d, *J* = 7.5 Hz, 1H), 6.81 (d, *J* = 8.7 Hz, 2H), 7.14 (dd, *J* = 8.7 Hz, 2H),7.90 (d, *J* = 8.4 Hz, 1H); ^13^C-NMR δ 28.0, 41.9, 55.1, 55.3, 83.7, 106.9, 114.1, 127.2, 131.1, 142.8, 151.5, 159.2, 192.4. HRMS (EI-ion trap) *m/z*: [M]^+^ calcd. for C_17_H_21_NO_4_, 303.1471; found 303.1471.

*N-Boc-2-(3,5-dimethoxylphenyl)-2,3-dihydro-4-pyridone* (**5Ai**). Employing General Procedure A and using *N*-Boc-4-pyridone (97 mg, 0.5 mmol), [RhCl(C_2_H_4_)_2_]_2_ (2.92 mg, 0.0075 mmol), BINAP (10.3 mg, 0.0165 mmol), ArZnCl (1.0 M in THF, 3.0 equiv, 1.5 mmol) and TMSCl (163 mg, 1.5 mmol) after purification by flash column chromatography (silica, 10%–20% ethyl acetate:hexanes, *v/v*) gave clear sticky oil **5Ai** (141 mg, 85%): IR (neat) 2975 (w), 2838 (w), 1719 (s), 1664 (s), 1592 (s), 1457 (m), 1420 (m), 1368 (m), 1288 (s), 1203 (m), 1144 (s), 1066 (m), 999 (m), 940 (w); 833 (m), 759 (m), 697 (m) cm^−1^; ^1^H-NMR (300 MHz, CDCl_3_) δ 1.46 (br s, 9H), 2.76 (d, *J* = 16.5 Hz, 1H), 3.11 (dd, *J* = 7.8, 16.5 Hz, 1H), 3.74 (s, 6H), 5.33 (d, *J* = 8.4 Hz, 1H), 5.58 (d, *J* = 7.8 Hz, 1H), 6.33 (s, 3H), 7.94 (d, *J* = 8.4 Hz, 1H); ^13^C-NMR (75 MHz, CDCl_3_) δ 28.0, 41.9, 55.3, 55.7, 83.8, 99.3, 104.1, 107.0, 125.3, 128.2, 129.0, 141.3, 142.9, 151.4, 161.1, 192.0. HRMS (EI-ion trap) *m/z*: [M]^+^ calcd for C_18_H_23_NO_5_, 333.1576; found 333.1578.

*N-Boc-2-(4-trifluoromethylphenyl)-2,3-dihydro-4-pyridone* (**5Ak**). Employing General Procedure A and using *N*-Boc-4-pyridone (97 mg, 0.5 mmol), [RhCl(C_2_H_4_)_2_]_2_ (2.92 mg, 0.0075 mmol), BINAP (10.3 mg, 0.0165 mmol), ArZnCl (1.0 M in THF, 1.5 equiv, 0.75 mmol) and TMSCl (163 mg, 1.5 mmol) after purification by flash column chromatography (silica, 10%–20% ethyl acetate:hexanes, *v/v*) gave white amorphous solid **5Ak** (80 mg, 47%): m.p. 78.1−79.5 °C; IR (neat) 3076 (w), 2980 (w), 2930 (w), 1728 (s), 1661 (s), 1604 (s)1474 (w), 1454 (m), 1394 (m), 1309 (s), 1258 (m), 1217 (m), 1147 (s), 1110 (s), 1069 (s), 1017 (m), 979 (w), 839 (m), 759 (m) cm^−1^; ^1^H-NMR (300 MHz, CDCl_3_) δ 1.42 (br s, 9H), 2.71 (d, *J* = 16.8 Hz, 1H), 3.14 (dd, *J* = 7.2, 16.8 Hz, 1H), 5.32 (d, *J* = 8.4 Hz, 1H), 5.66 (d, *J* = 7.2 Hz, 1H), 7.28 (d, *J* = 8.1 Hz, 2H), 7.53 (d, *J* = 8.1 Hz, 2H),7.92 (d, *J* = 8.4 Hz, 1H); ^13^C-NMR (75 MHz, CDCl_3_) δ 28.0, 41.6, 55.4, 84.2, 107.2, 125.87, 125.93, 126.2, 130.1, 130.5, 142.8, 151.2, 191.3. ^19^F-NMR (282 MHz, CDCl_3_) δ −62.7; HRMS (EI-ion trap) *m/z*: [M]^+^ calcd for C_17_H_18_NO_3_F_3_, 341.1239; found 341.1242.

*N-Boc-2-(3,5-ditrifluoromethylphenyl)-2,3-dihydro-4-pyridone* (**5Al**). Employing General Procedure A and using *N*-Boc-4-pyridone (97 mg, 0.5 mmol), [RhCl(C_2_H_4_)_2_]_2_ (2.92 mg, 0.0075 mmol), BINAP (10.3 mg, 0.0165 mmol), ArZnCl (1.0 M in THF, 1.5 equiv, 0.75 mmol) and TMSCl (163 mg, 1.5 mmol) after purification by flash column chromatography (silica, 10%–20% ethyl acetate:hexanes, *v/v*) gave yellow sticky oil **5Al** (84 mg, 41%): IR (neat) 2981 (w), 1725 (m), 1671 (m), 1601 (m), 1459 (w), 1417 (w), 1372 (w), 1303 (m), 1275 (s), 1213 (m), 1125 (s), 1014 (m), 897 (m), 846 (m), 766 (m) cm^−1^; ^1^H-NMR (300 MHz, CDCl_3_) δ 1.41 (br s, 9H), 2.73 (d, *J* = 16.5 Hz, 1H), 3.15 (dd, *J* = 7.8, 16.5 Hz, 1H), 5.34 (d, *J* = 8.4 Hz, 1H), 5.69 (d, *J* = 7.8 Hz, 1H), 7.57 (s, 2H), 7.90 (s, 1H), 7.92 (d, *J* = 8.4 Hz, 1H); ^13^C-NMR (75 MHz, CDCl_3_) δ 27.9, 41.2, 55.0, 84.8, 107.4, 117.6, 121.2, 122.2, 124.8, 126.1, 132.2, 132.6, 141.7, 142.6, 150.9, 190.6. ^19^F-NMR (282 MHz, CDCl_3_) δ −63.0; HRMS (EI-ion trap) *m/z*: [M]^+^ calcd for C_18_H_17_NO_3_F_6_, 409.1113; found 409.1119.

## 4. Conclusions

We have successfully developed rhodium(I)-complexes-catalyzed 1,4-conjugate additions of arylzinc chlorides to *N*-Boc-4-pyridone in the presence of chlorotrimethylsilane (TMSCl). A combination of [RhCl(C_2_H_4_)_2_]_2_ and BINAP was determined to be the most effective catalyst for 1,4-conjugate addition of arylzinc chlorides to *N*-Boc-4-pyridones. We also demonstrated that this reaction is compatible with a broad scope of substrates with both electron-withdrawing and electron-donating substituents on the aromatic ring to afford 1,4-adducts 2-substituted-2,3-dihydropyridones in high yields. Excellent ee can be achieved when (*S*)-BINAP is used as the chiral ligand. These 1,4-adducts are versatile intermediates that can be utilized for the synthesis of medicinally important *N*-heterocycles such as pyridines, piperidones, piperidines, indolizidenes, and quinolizidenes.

## Supplementary Materials

Supplementary materials are available online.

## Abbreviations

Boc	tert-Butyloxycarbonyl
C-C	Carbon carbon
BINAP	2,2’-bis(diphenylphosphino)-1,1’-binaphthyl
