# Supplementary material for: Rhodium(I)-Complexes Catalyzed 1,4-Conjugate Addition of Arylzinc Chlorides to N-Boc-4-pyridone
Source: Molecules. 2017 May 1;22(5):723. doi: 10.3390/molecules22050723 (PMC6154701; doi:10.3390/molecules22050723)

Supporting Information

Rhodium(I)-Complexes Catalyzed 1,4-Conjugate Addition of Arylzinc Chlorides to *N*-Boc-4-Pyridone

Fenghai Guo [1, *], Matthew A. McGilvary [1], Malcolm C. Jeffries [1], Briana N. Graves [1], Shekinah A. Graham [1], Yuelin Wu [2]

[1] Department of Chemistry, Winston-Salem State University; guof@wssu.edu; Tel.: +01-336-750-3158

[2] School of Chemical and Environmental Engineering, Shanghai Institute of Technology, Shanghai, P. R. China 201418;

* Correspondence: WBA 311, 601 S. Martin Luther King Jr. Dr., Winston-Salem, North Carolina, USA 27110; guof@wssu.edu; Tel.: +01-336-750-3158

Contents **Page**

^{1}H, ^{13}C NMR of compounds **5Aa, 5Ad, 5Af-Ag, 5Ai, 5Ak-Al** S1-S12, S14-15

^{19}F NMR of compounds **5Ak-Al** S13 and S16

Guo-4-Me
wfu_PROTON CDCl3 /opt/topspin guof 30

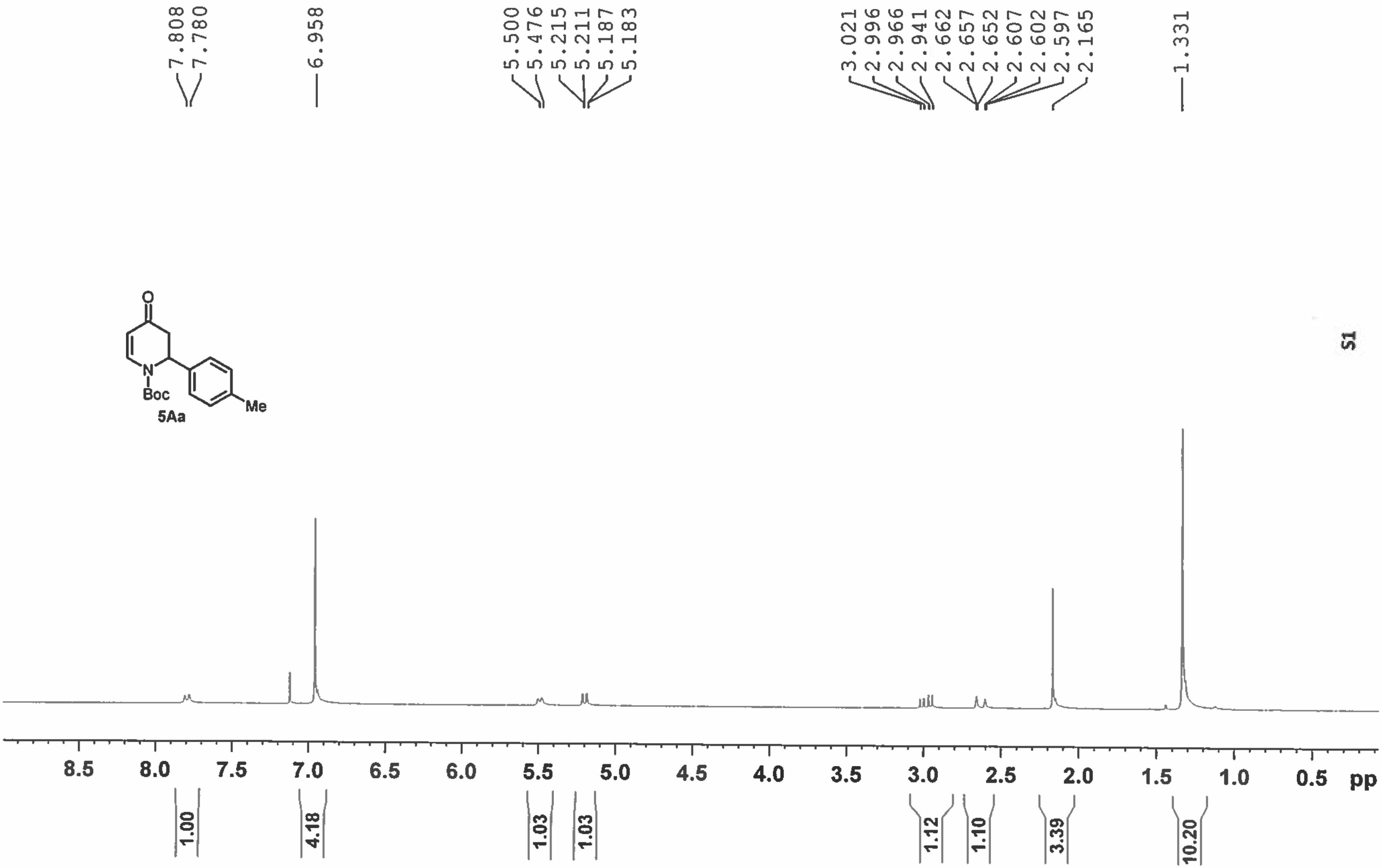

Guo4-Me
wfu_C13CPD_128 CDCl3 /opt/topspin guof 30

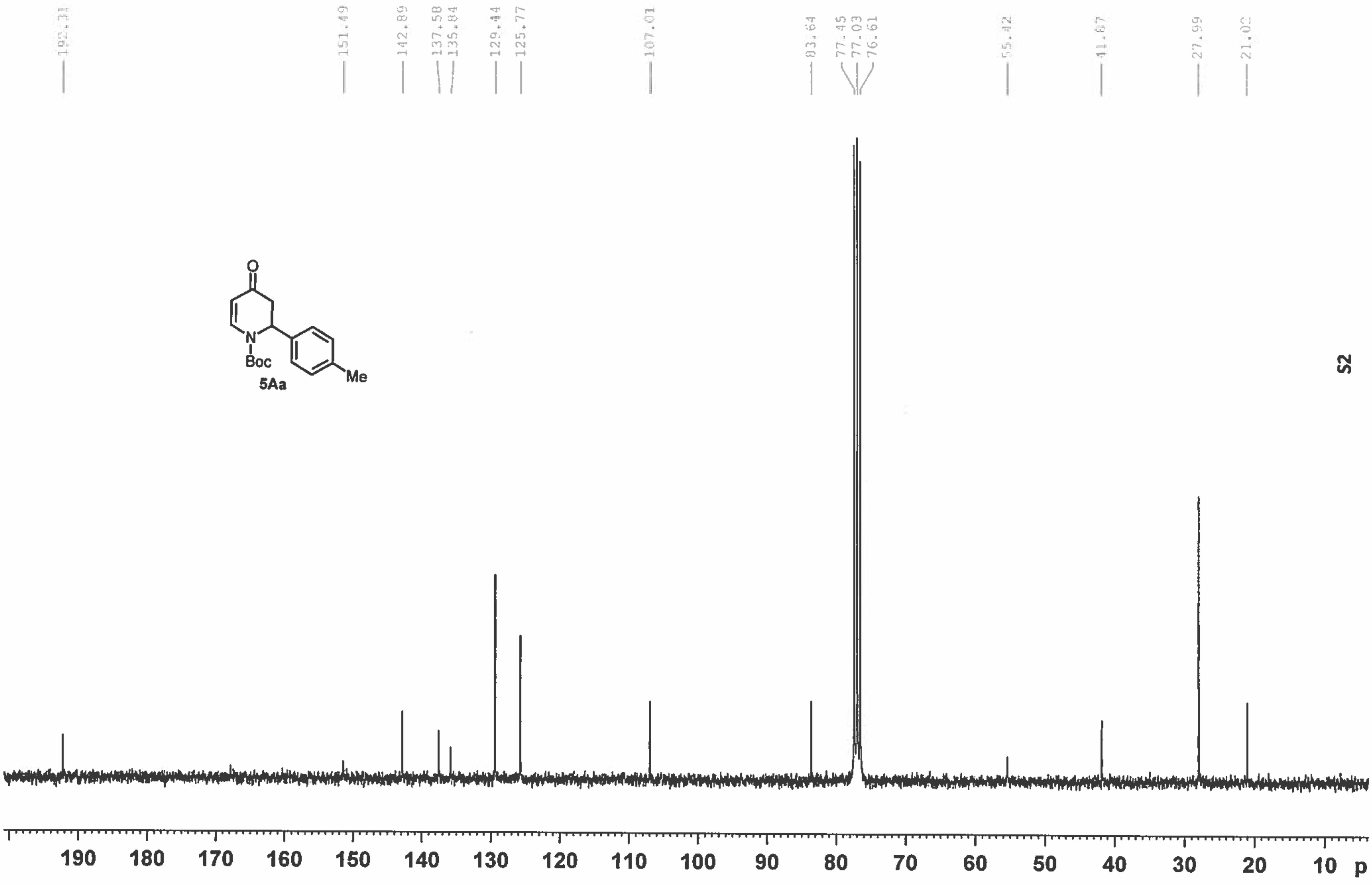

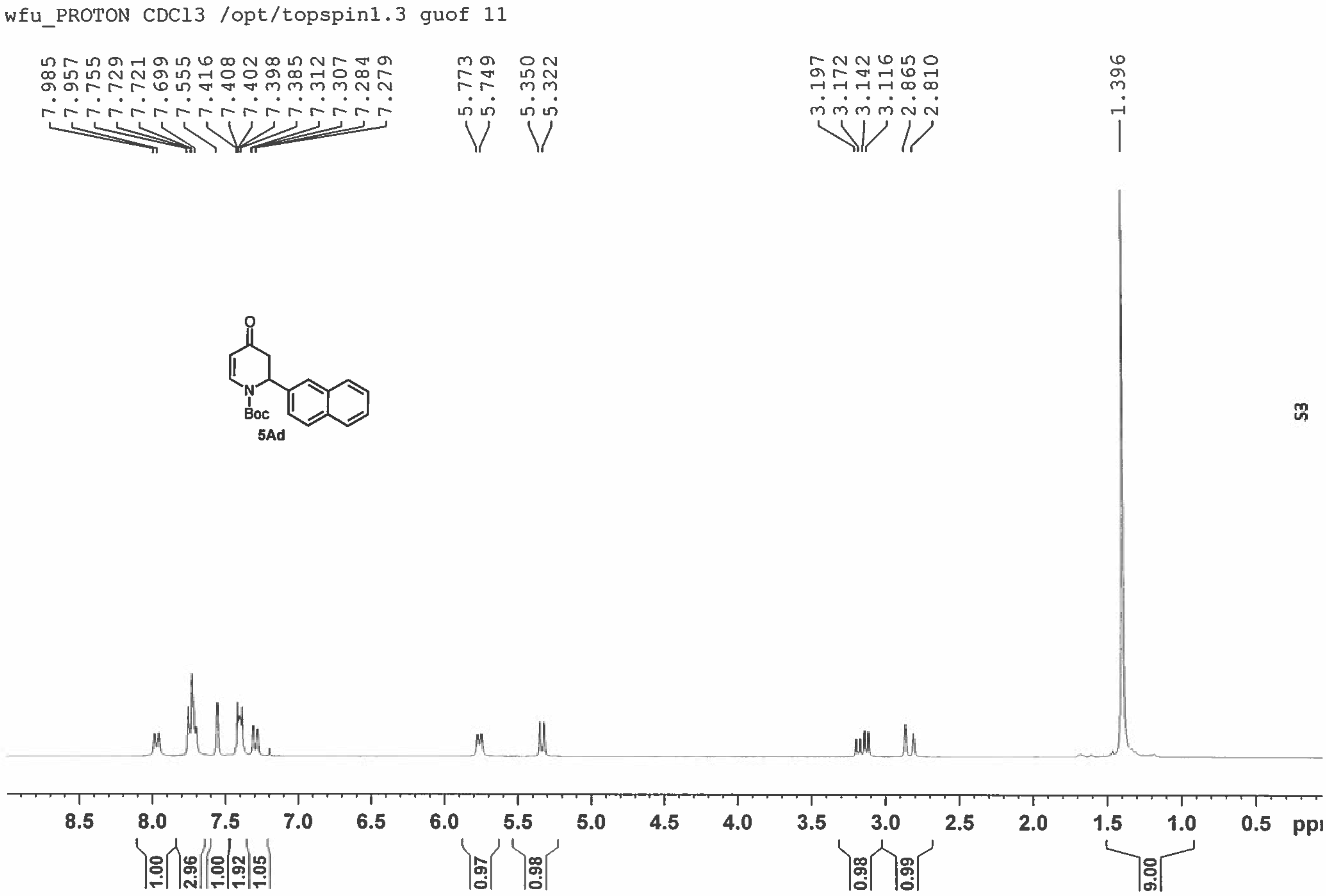
wfu_PROTON CDCl3 /opt/topspin1.3 guof 11
7.985
7.957
7.755
7.729
7.721
7.699
7.555
7.416
7.408
7.402
7.398
7.385
7.312
7.307
7.284
7.279
5.773
5.749
5.350
5.322
3.197
3.172
3.142
3.116
2.865
2.810
1.396
O
N
Boc
5Ad
8.5
8.0
7.5
7.0
6.5
6.0
5.5
5.0
4.5
4.0
3.5
3.0
2.5
2.0
1.5
1.0
0.5
ppm
1.00
2.96
1.00
1.92
1.05
0.97
0.98
0.98
0.99
9.00

wfu_C13CPD_128 CDCl3 /opt/topspin1.3 guof 11
Guo-2-nap

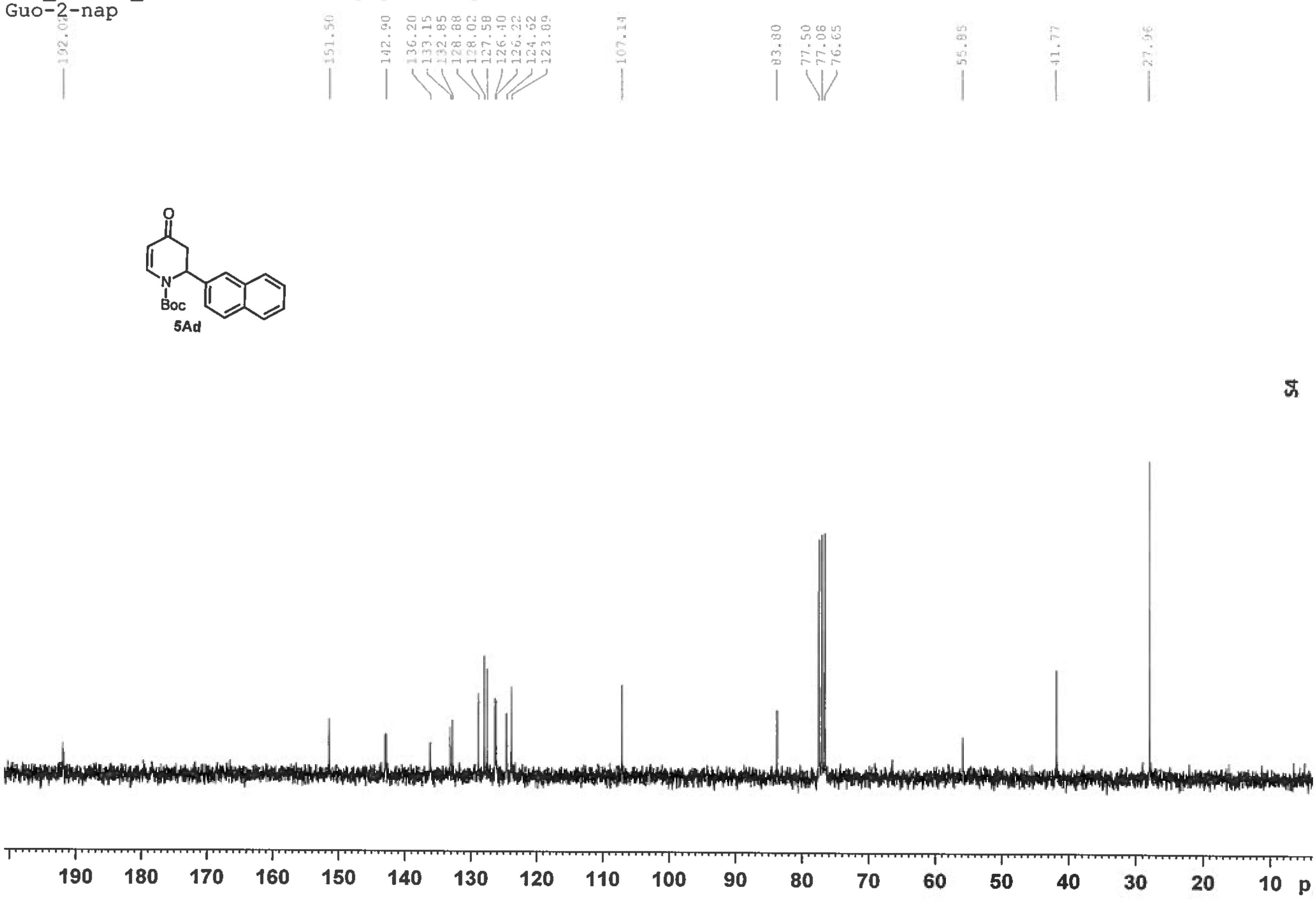

wfu_PROTON CDCl3 /opt/topspin1.3 guof 14
3,5-dimethyl

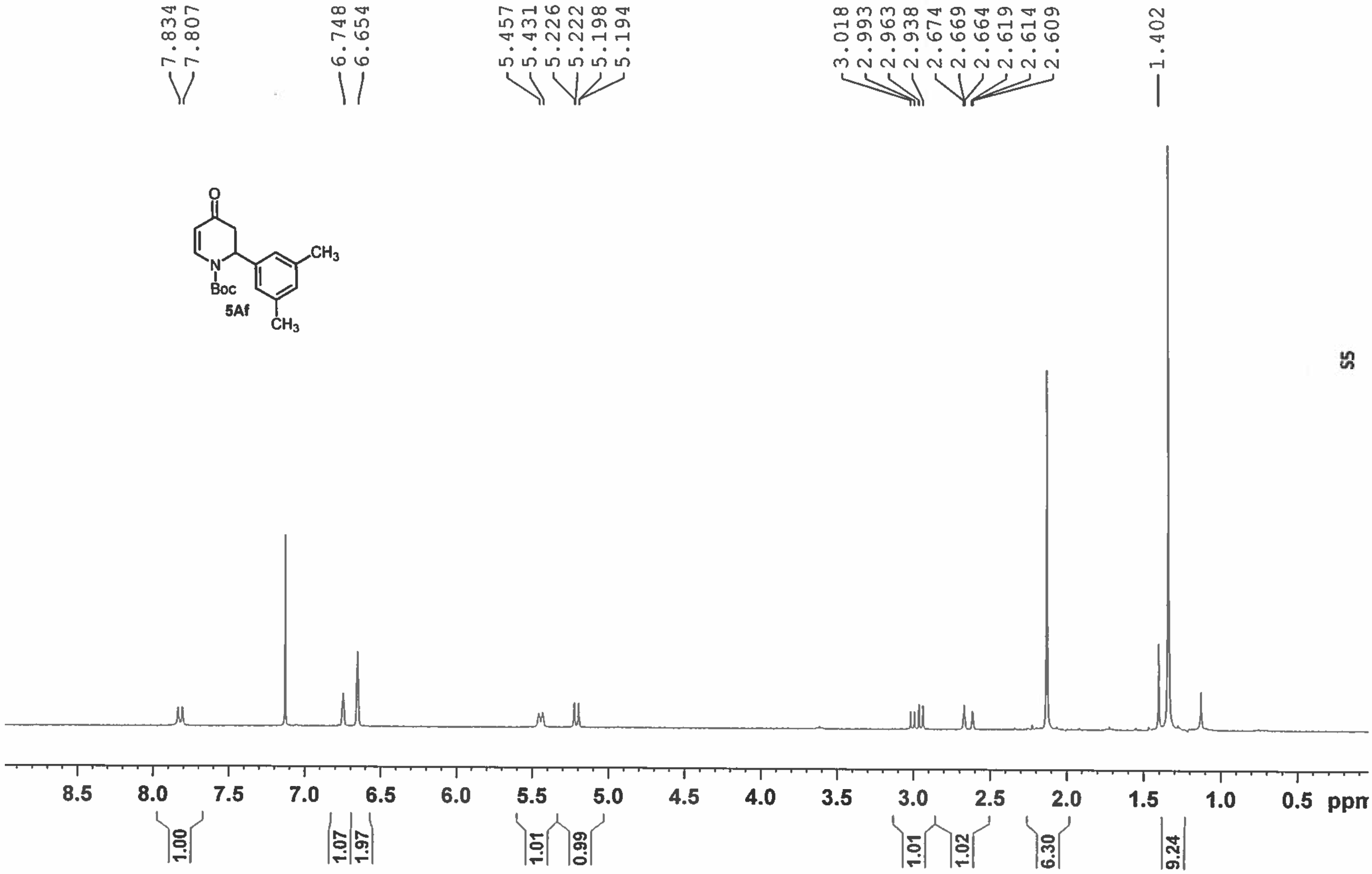

Guo-3,5-dimethyl
wfu_C13CPD_128 CDCl3 /opt/topspin guof 41

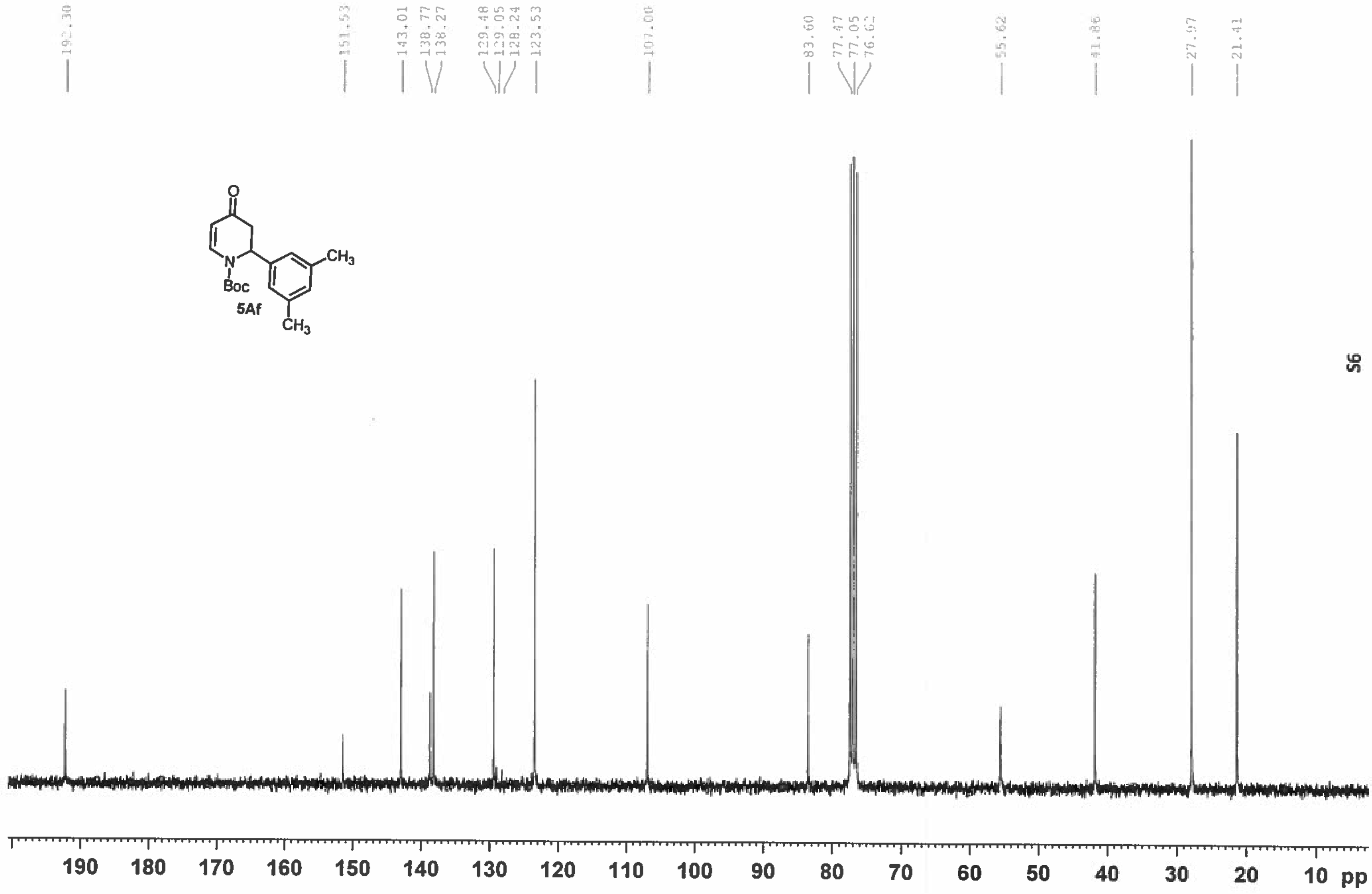

Guo-4-OMe
wfu_PROTON CDCl3 /opt/topspin guof 31

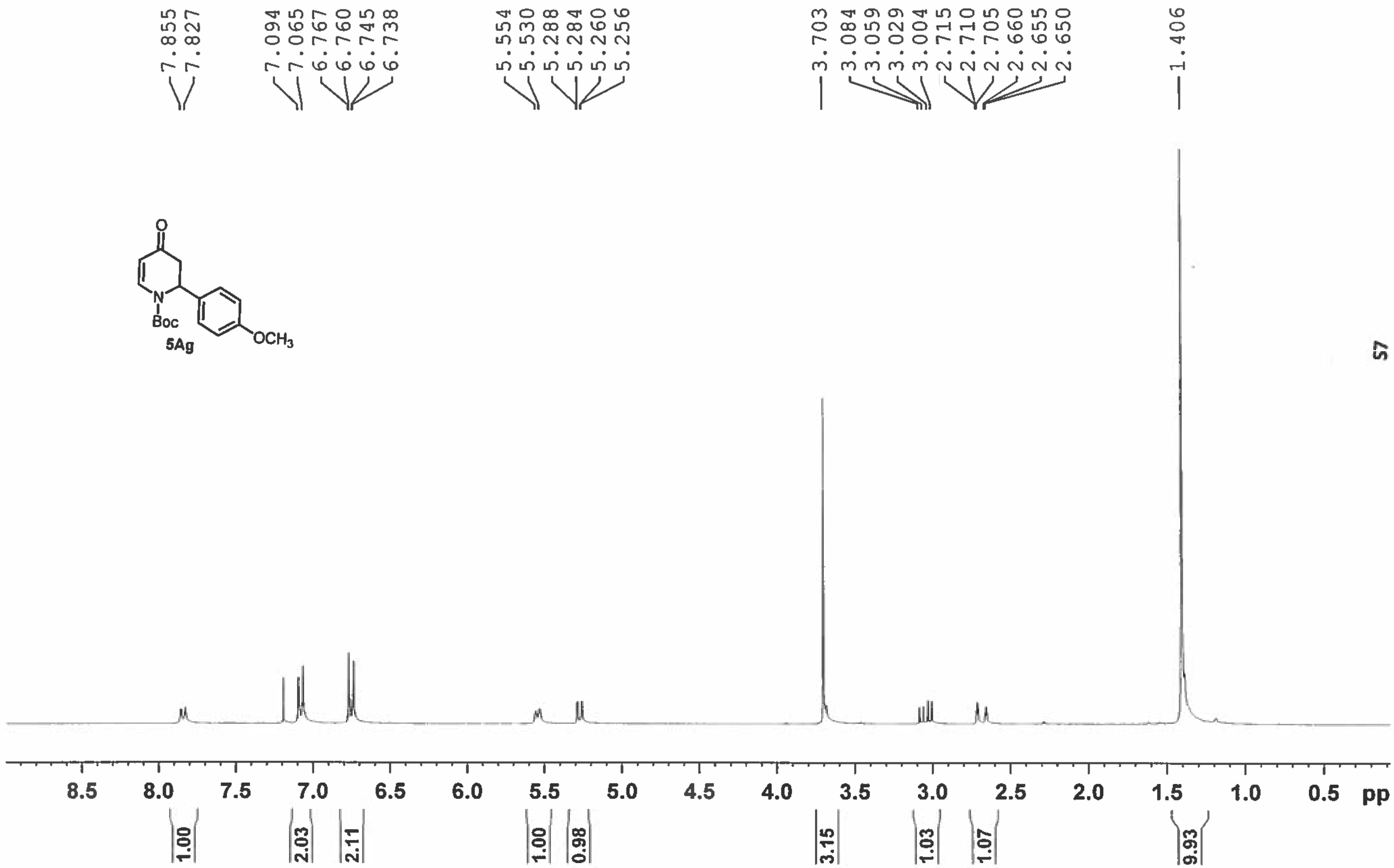

Guo-4-OMe
wfu_C13CPD_128 CDCl3 /opt/topspin guof 31

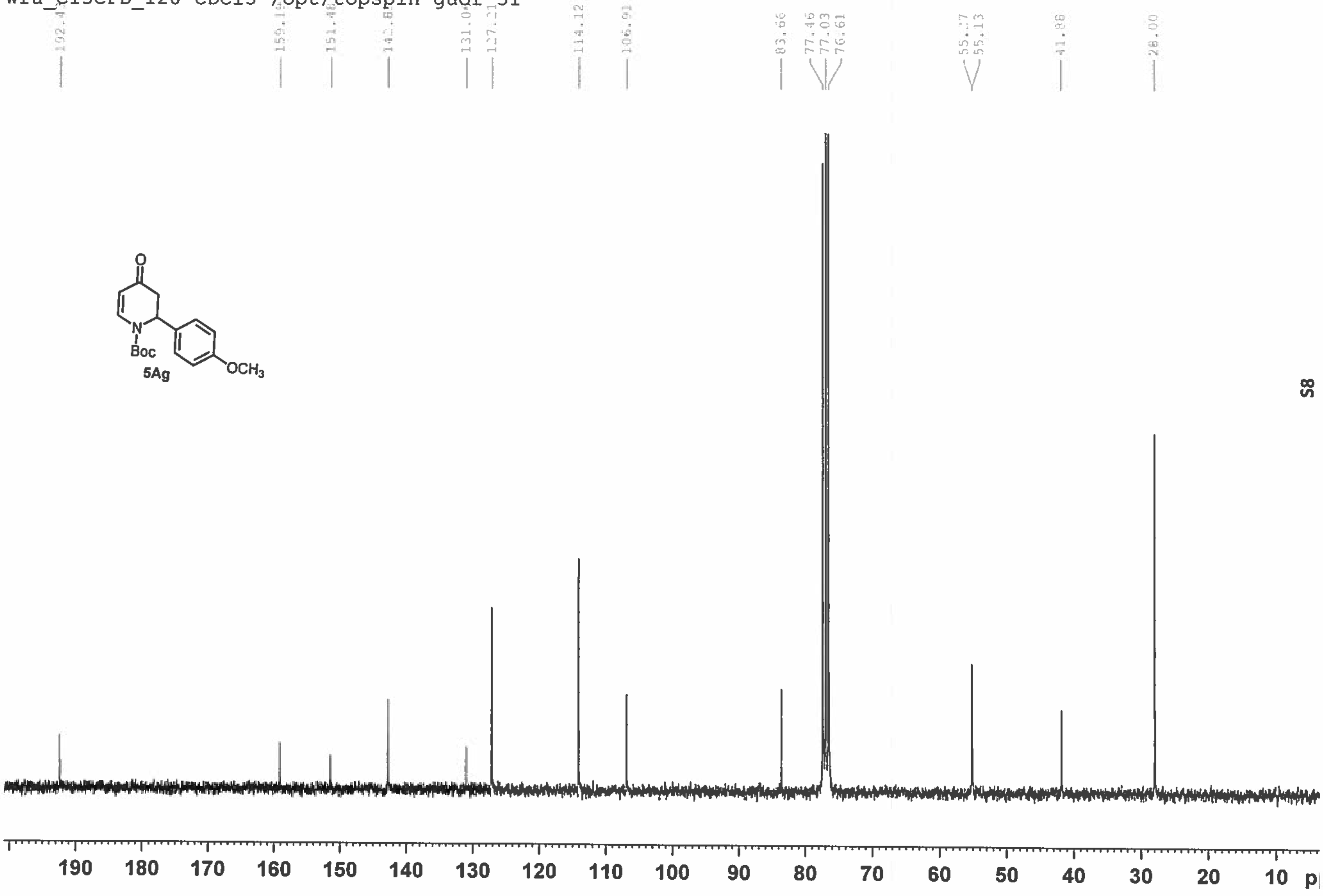

Guo-dimethoxyl
wfu_PROTON CDCl3 /opt/topspin guof 42

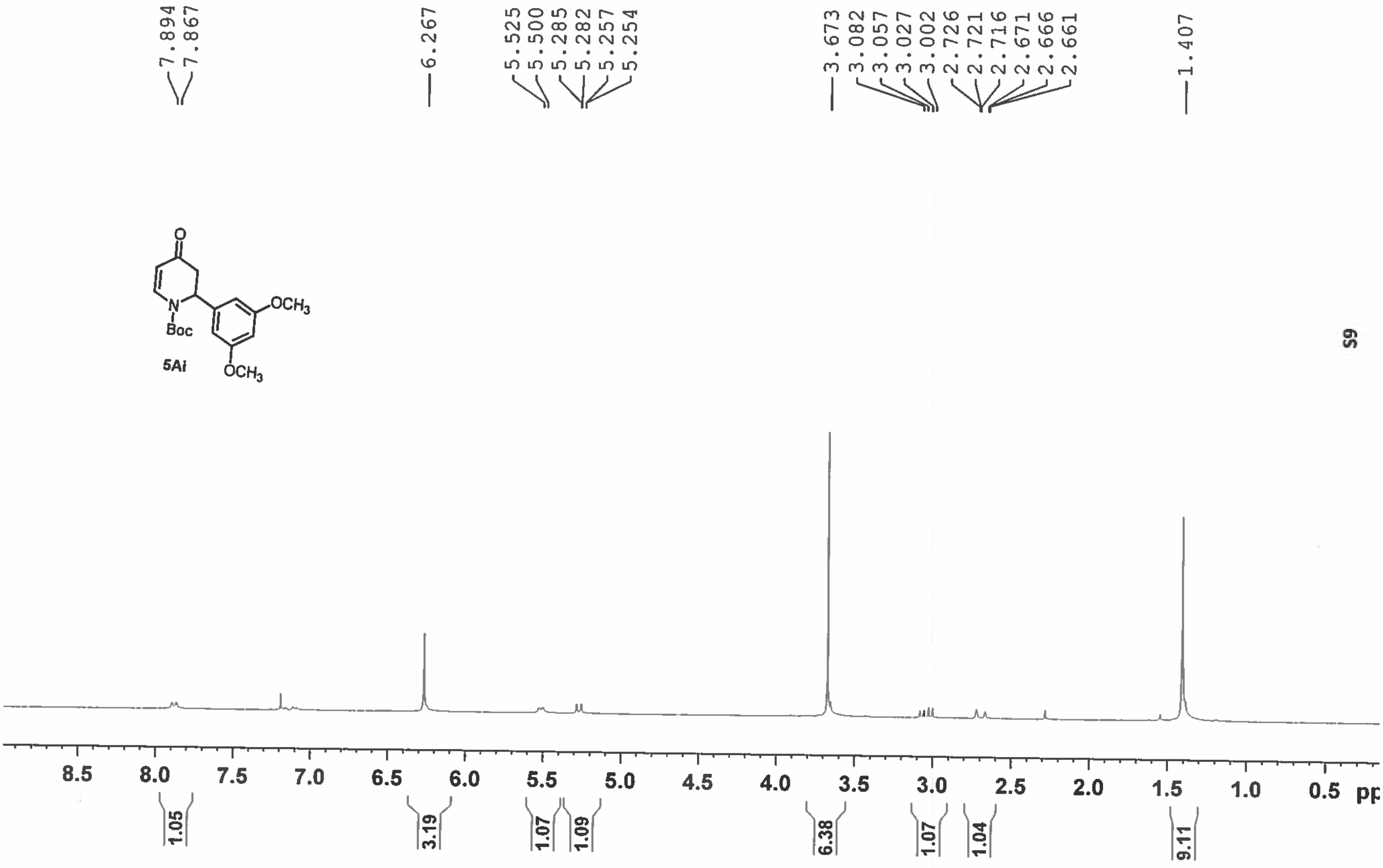

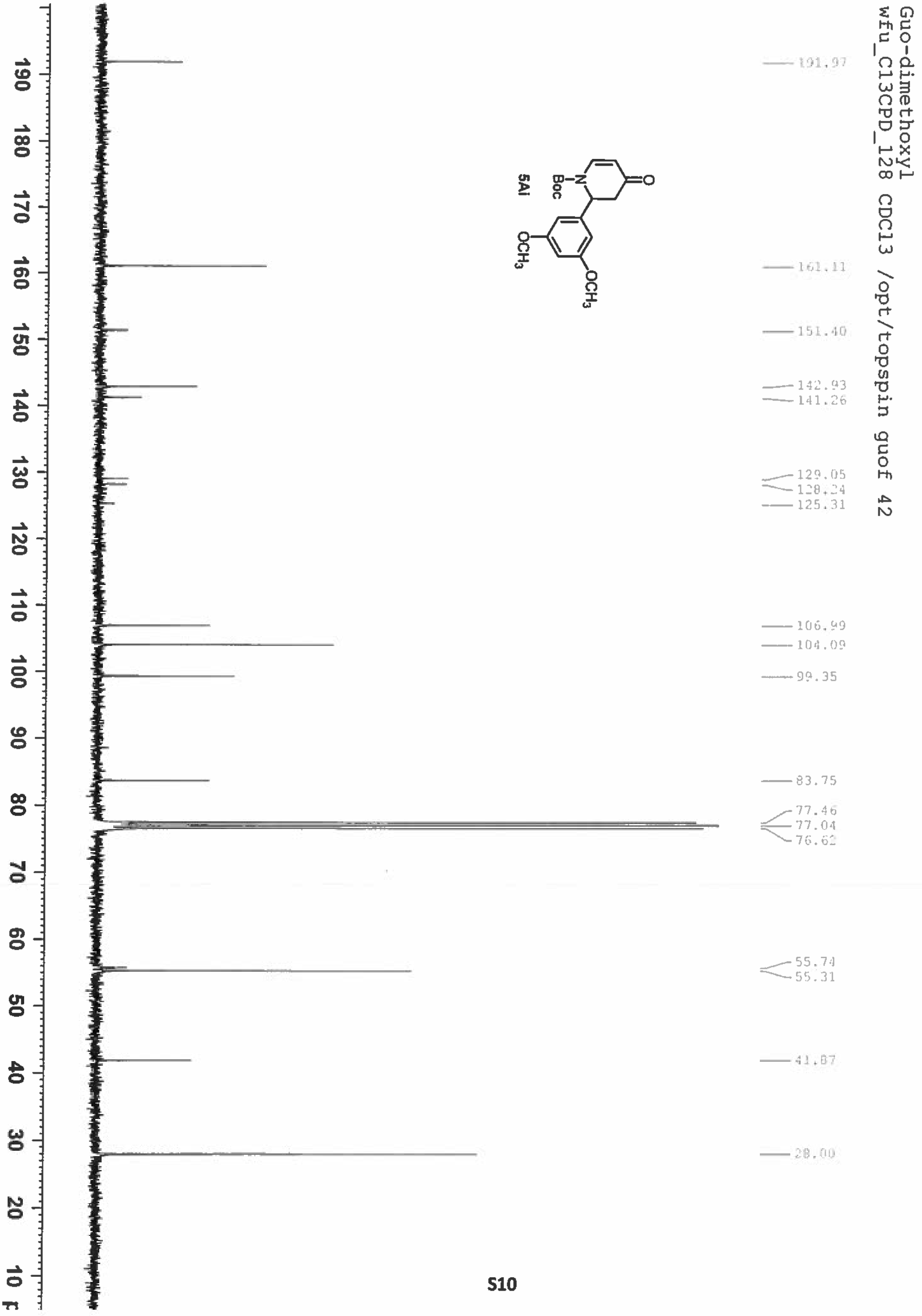

Guo-dimethoxyl
wfu_C13CPD_128 CDCl3 /opt/topspin guof 42
191.97
161.11
151.40
142.93
141.26
129.05
128.24
125.31
106.99
104.09
99.35
83.75
77.46
77.04
76.62
55.74
55.31
41.87
28.00
O
N
Boc
OCH3
OCH3
5Ai
190
180
170
160
150
140
130
120
110
100
90
80
70
60
50
40
30
20
10

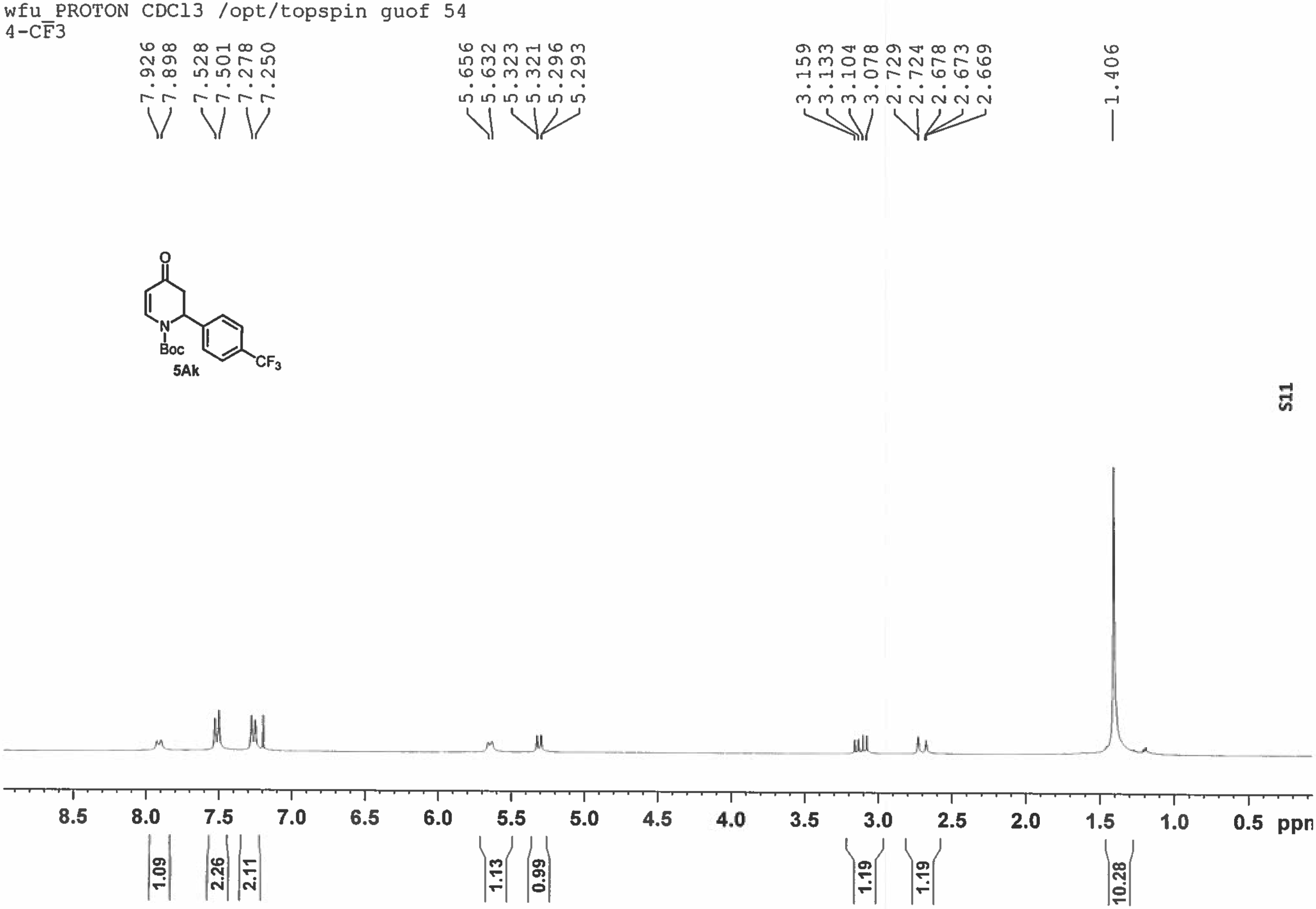

wfu_PROTON CDCl3 /opt/topspin guof 54
4-CF3
7.926
7.898
7.528
7.501
7.278
7.250
5.656
5.632
5.323
5.321
5.296
5.293
3.159
3.133
3.104
3.078
2.729
2.724
2.678
2.673
2.669
1.406
Boc
5Ak
CF_3
8.5
8.0
7.5
7.0
6.5
6.0
5.5
5.0
4.5
4.0
3.5
3.0
2.5
2.0
1.5
1.0
0.5 ppm
1.09
2.26
2.11
1.13
0.99
1.19
1.19
10.28

Guo-4-CF3
wfu_C13CPD_128 CDCl3 /opt/topspin guof 54

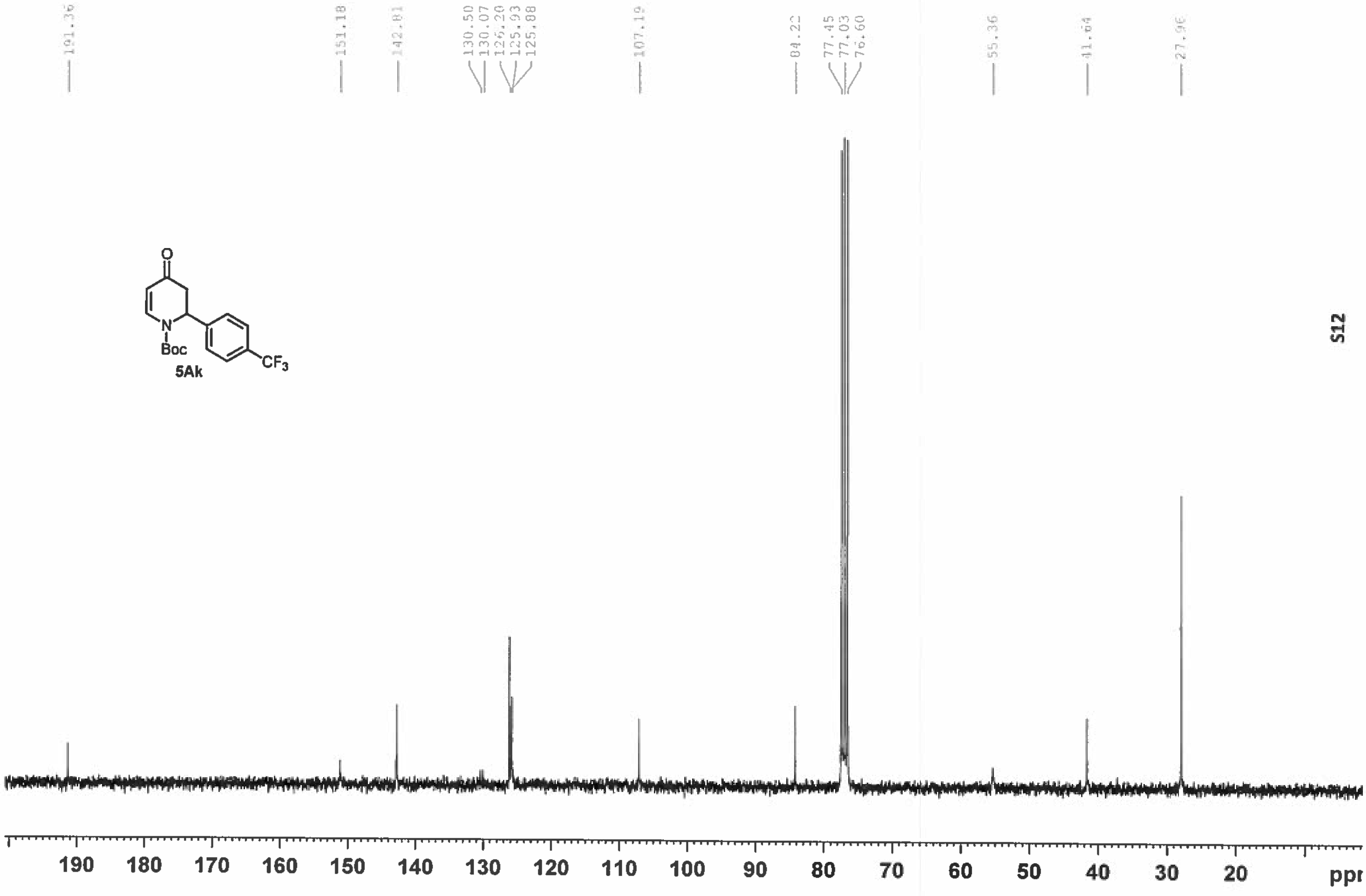

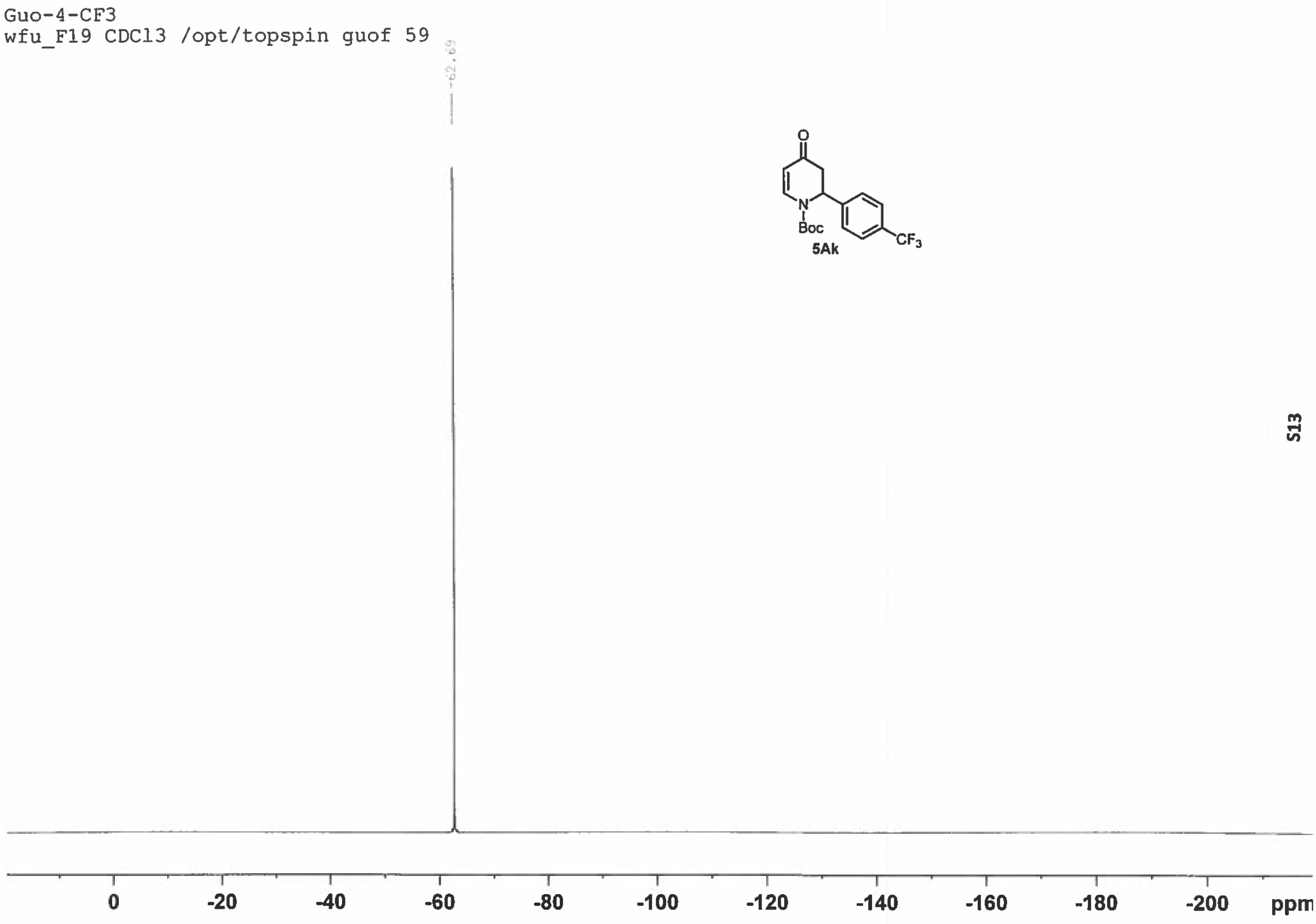
Guo-4-CF3
wfu_F19 CDCl3 /opt/topspin guof 59
-62.69
O
N
Boc
5Ak
CF3
0
-20
-40
-60
-80
-100
-120
-140
-160
-180
-200
ppm

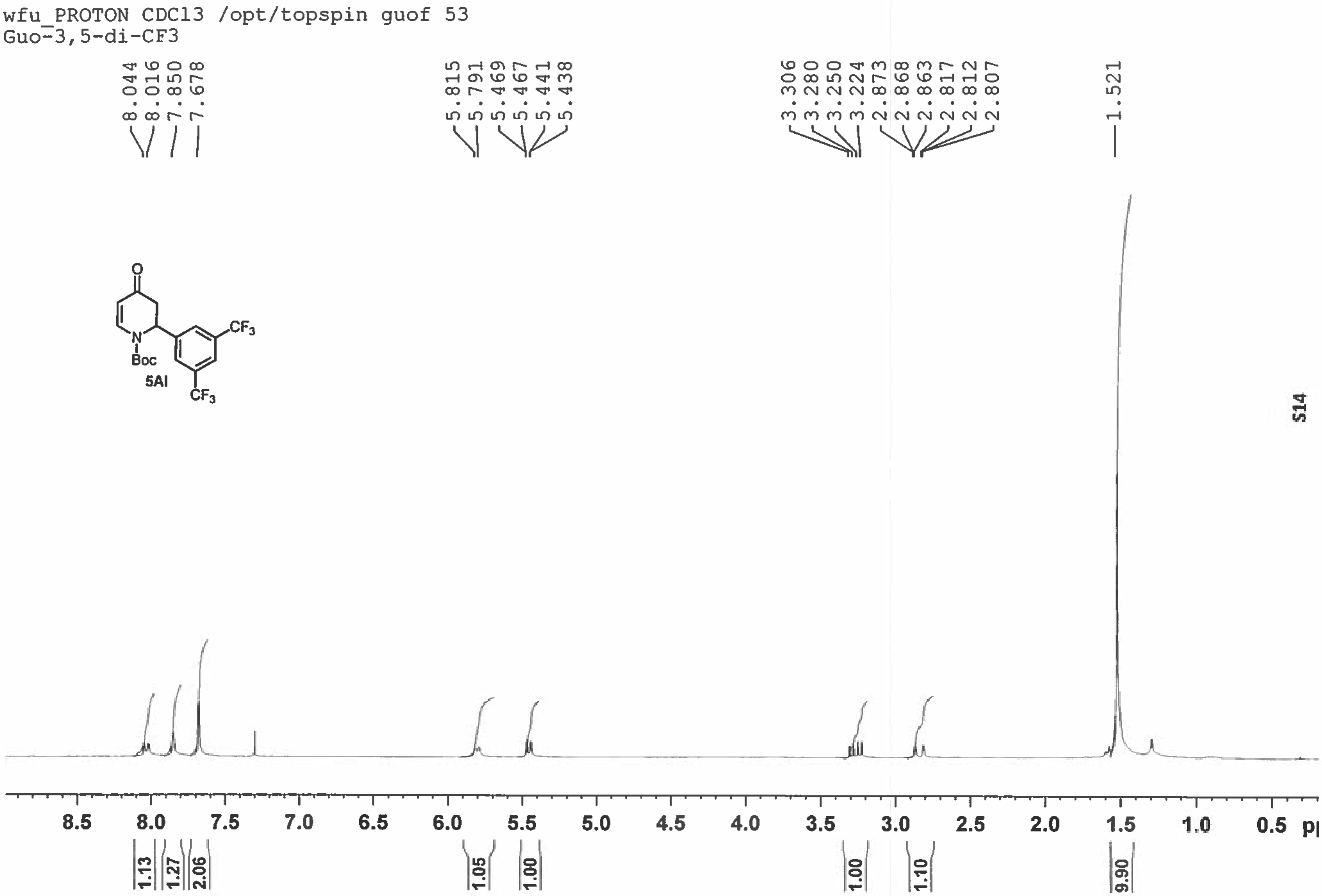
wfu_PROTON CDCl3 /opt/topspin guof 53
Guo-3,5-di-CF3
8.044
8.016
7.850
7.678
5.815
5.791
5.469
5.467
5.441
5.438
3.306
3.280
3.250
3.224
2.873
2.868
2.863
2.817
2.812
2.807
1.521
O
N
Boc
CF3
CF3
5AI
8.5
8.0
7.5
7.0
6.5
6.0
5.5
5.0
4.5
4.0
3.5
3.0
2.5
2.0
1.5
1.0
0.5
1.13
1.27
2.06
1.05
1.00
1.00
1.10
9.90

Guo-3,5-di-CF3
wfu_C13CPD_128 CDCl3 /opt/topspin guof 53

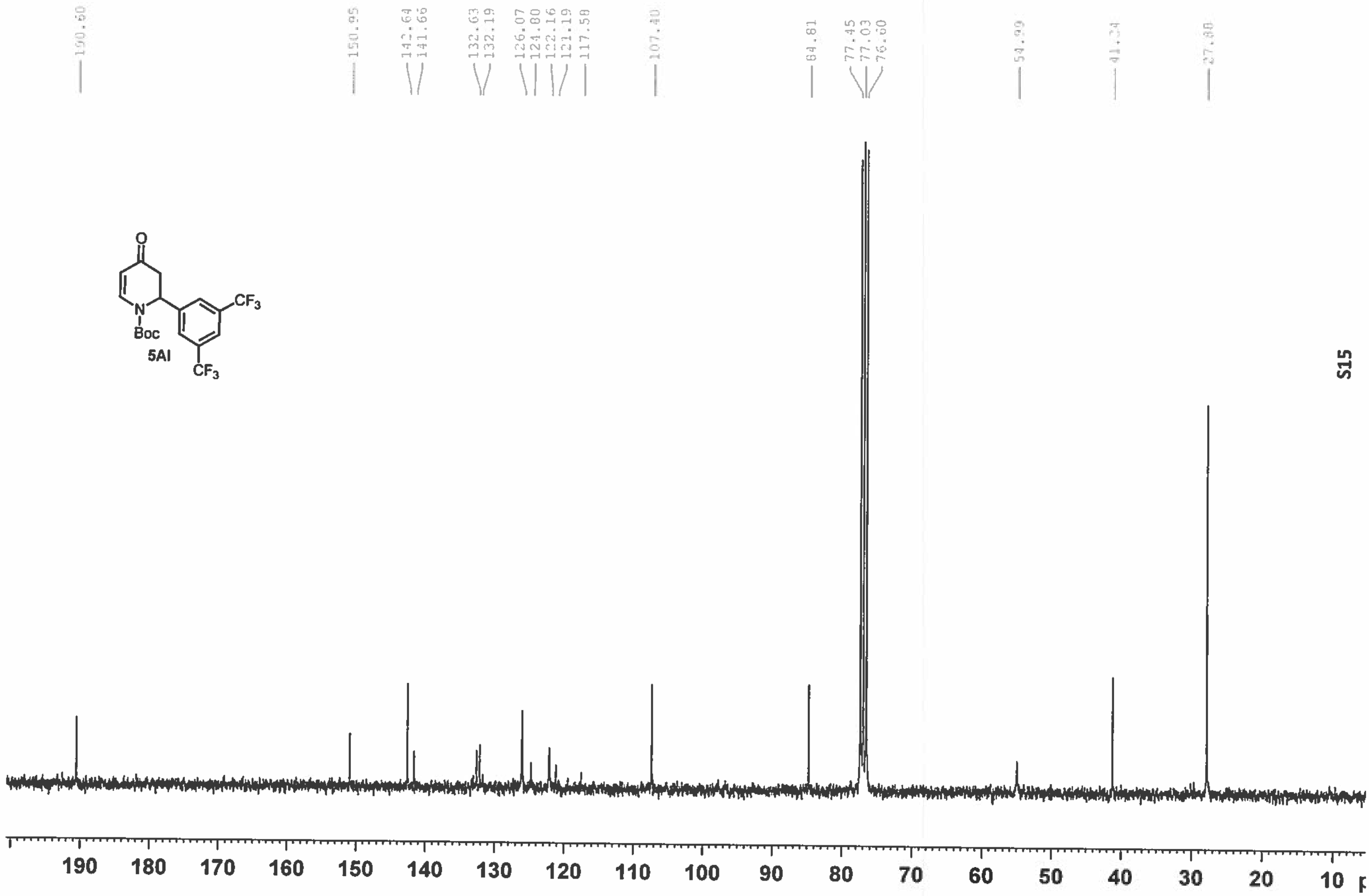

Guo-3,5-di-CF3
wfu_F19 CDCl3 /opt/topspin guof 58

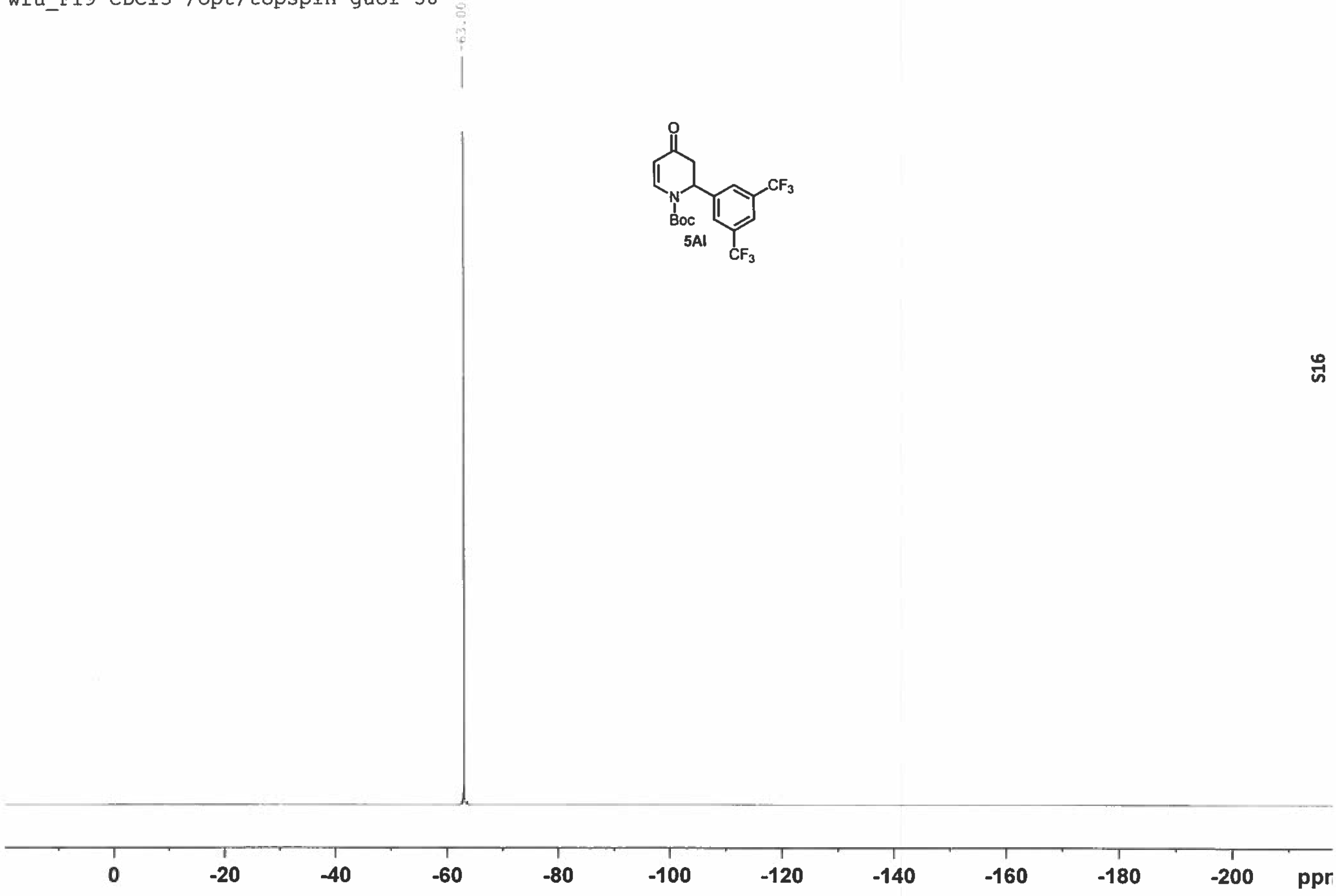

Supplement: Supplementary file 1 [file molecules-22-00723-s001.pdf]
